# Late diagnosis among our ageing HIV population: a cohort study

**DOI:** 10.7448/IAS.17.4.19692

**Published:** 2014-11-02

**Authors:** Sarah Mensforth, Lisa Goodall, Neena Bodasing, James Coultas

**Affiliations:** 1 Stoke and Staffordshire Partnership Trust, Cobridge Sexual Health, Stoke-on-Trent, UK; 2 Infectious Diseases, University Hospital of North Staffordshire, Stoke-on-Trent, UK; 3 University Hospital of North Staffordshire, Keele University School of Medicine, Stoke-on-Trent, UK

## Abstract

**Introduction:**

With the advent of combined antiretroviral therapy (cART), more people infected with HIV are living into older age; 22% of adults receiving care in the UK are aged over 50 years [1]. Age influences HIV infection; the likelihood of seroconversion illness, mean CD4 count and time from infection to development of AIDs defining illnesses decreases with increasing age. A UK study estimates that half of HIV infections in persons over 50 years are acquired at an age over 50 [2]. Studies exploring sexual practices in older persons have repeatedly shown that we cannot assume there is no risk of STI and HIV infection [3,4]. Physicians should be alert to risk of HIV even in the older cohort, where nearly half diagnoses are made late [2]. Local audit has demonstrated poor testing rates in the over 50's on the Acute Medical Unit. Late diagnosis (CD4<350) results in poorer outcomes and age confounds further; older late presenters are 2.4 times more likely to die within the first year of diagnosis than younger counterparts [2].

**Materials and Methods:**

A retrospective case notes review was conducted of all patients aged 60 years and over attending HIV clinic in the last 2 years. Outcomes audited included features around diagnosis; age, presentation, missed testing opportunities and CD4 count at diagnosis.

**Results:**

Of the current cohort of 442 patients, 34 were over 60 years old (8%). Age at diagnosis in this group ranged from 36 to 80 years, mean 56.6 years. Presentation triggers included opportunistic infections or malignancies (n=10), constitutional symptoms (n=6), diagnosis of another STI (n=4), seroconversion illness (n=2), partner status (n=3). Eight patients were diagnosed through asymptomatic screening at Sexual Health. We identified missed opportunities in five patients who were not tested despite diagnoses or symptoms defined as clinical indicators for HIV. Half of older patients had a CD4 count of <200 at diagnosis.

**Conclusions:**

It is imperative that general medical physicians and geriatricians are alert to enquiring about risk and testing for HIV where clinical indicators are present, irrespective of age. The oldest patient in the cohort was diagnosed with HIV aged 80 years. All patients with missed opportunities for testing were over 47 years old.

## References

[1] Health Protection Agency. HIV in the United Kingdom (2012). http://www.hpa.org.uk/Publications/InfectiousDiseases/HIVAndSTIs/1211HIVintheUK2012/.

[2] Delpech VC, Brown AE, Rice BD, Smith RD (2010). HIV transmission and high rates of late diagnoses among adults aged 50 years and over. AIDS.

[3] The National Survey of Sexual Attitudes and Lifestyles (2012). http://www.natsal.ac.uk/natsal-3.

[4] Bodley-Tickell AT, Olowokure B, Bhaduri S, White DJ, Ward D, Ross JD (2008). Trends in sexually transmitted infections (other than HIV) in older people: analysis of data from an enhanced surveillance system. Sex Transm Infect.

